# Infection Preventionist Work Profiles and Organizational Capacity for Policy Compliance in Georgia Nursing Homes

**DOI:** 10.1093/geroni/igaf122.1158

**Published:** 2025-12-31

**Authors:** Yu Jin Kang, Jacqueline Jordan, Monika Pogorzelska-Maziarz, Jennifer Morgan, Dawn Aycock

**Affiliations:** Georgia State University, Atlanta, Georgia, United States; Georgia State University, Atlanta, Georgia, United States; Villanova University, Villanova, Pennsylvania, United States; Georgia State University, Atlanta, Georgia, United States; Georgia State University, Atlanta, Georgia, United States

## Abstract

**Objective:**

Infection Preventionists (IPs) play a critical role in ensuring compliance with infection prevention and control (IPC) policies in nursing homes (NHs), yet workforce stability remains a challenge. This pilot study explored IP work profiles and organizational capacity for IPC policy compliance in Georgia NHs.

**Methods:**

A multi-method approach was used to survey IPs and conduct interviews with NH administrators (Feb-Dec, 2024). Recruitment was conducted through the Georgia Health Care Association, via phone and email, using snowball and purposive sampling. Work profiles were assessed via published survey tools, while facilitators and barriers to policy compliance were examined using Packard’s organizational change framework. Descriptive statistics summarized survey data, and deductive content analysis was applied to the interview narrative.

**Results:**

Six survey responses were collected. Most IPs were registered nurses (83%) with dual roles in nursing, staff education, or employee health. Over half (67%) had more than five years of experience, though half had been at their current facility for less than a year. All received state or local IPC training, and two were board-certified. Half worked in non-profit facilities. All reported multiple IP turnovers within the past three years. Facilitators from one administrator interview included “supportive leadership,” “organizational resources,” and “policy change pressures,” while barriers included “government-industry miscommunication” and “high IP turnover.”

**Conclusions:**

High IP turnover, possibly due to workload and pressure, and communication gaps hinder policy compliance. Strengthening workforce stability, government-industry collaboration, and structured IP competency training may reduce turnover. These findings can inform future research using a larger sample.

